# Congenital Cholesteatoma in A Case of Congenital Aural Atresia: A Case Report

**DOI:** 10.31729/jnma.5199

**Published:** 2020-12-31

**Authors:** Poonam Aggarwal, Pabina Rayamajhi

**Affiliations:** 1 Department of ENT and Head & Neck Surgery, TU Teaching Hospital, Maharajgunj Medical Campus, Institute of Medicine, Kathmandu, Nepal

**Keywords:** *canal atresia*, *congenital cholesteatoma*, *facial paresis*, *modified radical mastoidectomy*

## Abstract

Congenital external canal atresia is one of the congenital ear anomalies that can occur in patients. Similarly, congenital cholesteatoma is also another congenital disease which is often diagnose in early adulthood. Both the above-mentioned diseases can occur independently but the presence of both these entities is a rare occurrence and needs a high degree of suspicion aided by computed tomography scan to make the diagnosis. We are presenting a case of sixteen-year-old patient who presented with unilateral ear anomaly, earache, facial palsy and postaural swelling and was diagnosed as right sided congenital aural atresia with congenital cholesteatoma. He was surgically managed with right-sided modified radical mastoidectomy with canaloplasty and closure of mastoid fistula under general anesthesia.

## INTRODUCTION

Congenital external auditory canal atresia is a relatively uncommon condition with the incidence of 1 in 10,000–20,000 live births commonly a unilateral occurrence.^1^ Congenital cholesteatoma, one of the rare diseases of temporal bone has been reported to be present in 4-7% of cases of congenital external auditory canal atresia.^2^ When canal atresia is associated with cholesteatoma, the patients present late with features like facial nerve palsy or hearing loss. It needs a high degree of clinical judgment aided by high-resolution computed tomography (CT) scan and magnetic resonance and imaging (MRI) to assess the atresia and extent of the disease.^1,2^

## CASE REPORT

We are reporting a case of a sixteen-year-old male patient with right-sided external auditory canal atresia and left-sided microtia since birth.

The patient presented to ENT-HNS outpatient clinic of Tribhuvan University Teaching Hospital, along with his guardian with a history of right side external auditory atresia and left-sided microtia by birth. He had progressive right-sided hearing loss since early childhood. But he had no complaints of ear discharge, earache etc. till recently in the last five month she developed right-sided earache, mild facial weakness associated with postauricular swelling for which he had undergone incision and drainage at the peripheral clinic before presenting to us.

His ENT examination revealed right-sided lower motor neuron type grade II facial paresis and external auditory canal atresia and non-discharging fistula in post aural region. He also had on left side microtia grade III. Tuning fork examination using 512 Hz tuning fork revealed Rinne negative on both the sides and Weber lateralized to the left side.

**Figure 1:**
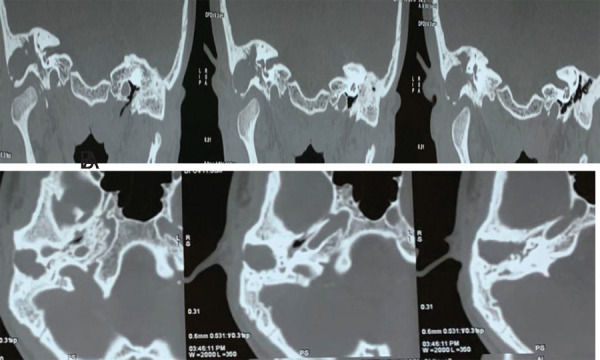
A, B. HRCT of the temporal bone-coronal and axial view.

Pure Tone Audiometry (PTA) revealed 67dB severe mixed type of hearing impairment on the right side and 61dB severe conductive type of hearing impairment in the left side. High-Resolution Computed Tomography (HRCT) temporal bone revealed sclerosis of the mastoid bone with loss of mastoid air cells due to chronic mastoiditis on the right side. Soft tissue lesion in the right middle ear, attic with the erosion of outer bony cortex. There was right-sided external auditory canal atresia also detected (Figure 1).

The patient was diagnosed as having right-sided canal atresia with congenital cholesteatoma and mastoid fistula. He was planned for right-sided mastoid exploration with canaloplasty and closure of the mastoid fistula. He was counselled regarding the hearing and facial nerve paresis.

**Figure 2:**
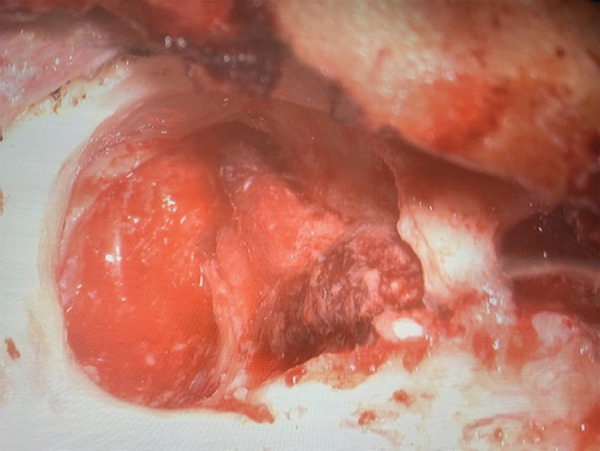
Intraoperative mastoid cavity and dehiscent vertical facial nerve.

Intraoperative finding showed complete atresia of the bony and cartilaginous part of the external auditory canal with the hypoplastic middle ear. Cholesteatoma was present in the attic, aditus, antrum, periantral, retrofacial, stapes, mesotympanum, sinus tympani, tip cells and eustachian tube. Granulation was present in antrum, periantral, retrofacial, mesotympanum, sinus tympani, eustachian tube. A segment of facial nerve was dehiscent from first genu to stylomastoid foramen with intact facial nerve sheath. The granulation tissue over the facial nerve was carefully dissected (Figure 2). Among the ossicles the malleus and incus were totally absent and stapessuperstructure was present.

**Figure 3:**
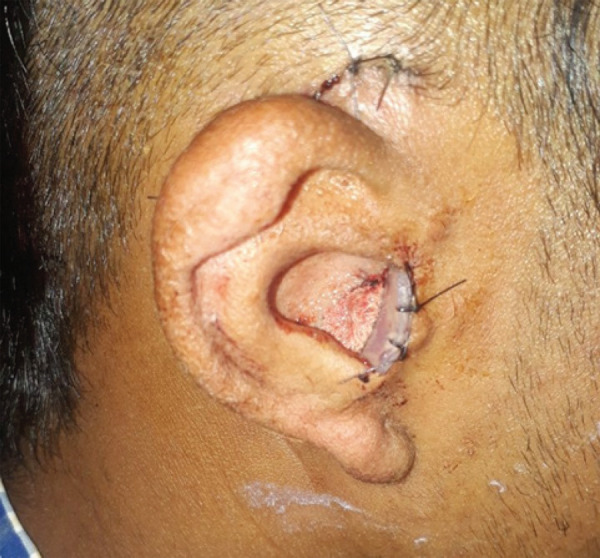
Postoperative stent in situ in the external auditory canal.

External auditory canal reconstruction was done by removal of the bone lateral to the middle ear space followed by meatoplasty of the cartilaginous portion. For tympanoplasty, temporalis fascia graftwas placed on the head of the stapes. Stent was kept in the reconstructed external auditory canal (Figure 3).

Postoperatively, the patient was kept in intravenous (IV) antibiotics, post aural suture removal was done on 6thday, the pack was removed on 10thday postoperatively, and silastic was removed 6 weeks postoperatively and meatus was well maintained. In follow up till eight weeks postoperatively, the status of the facial nerve was status quo. No intracranial or other post-operative complications were observed after surgery.

## DISCUSSION

The diagnosis of congenital cholesteatoma with canal atresia is challenging. Although rare, the incidence of congenital cholesteatoma is higher in ears with congenital aural atresia.^3^

In this case, the patient has congenital cholesteatoma of the temporal bone. The disease described as congenital when it manifests itself without a past history of trauma or infection with intact and located behind the tympanic membrane.^3^ Our case, presented with hearing loss, earache, facial paresis, postaural swelling after earache and had undergone incision and drainage, most likely he must have developed mastoiditis and mastoid abscess. Hidaka, et al.^4^ had reported a case of congenital cholesteatoma of the mastoid region in a sixty-five years old man that presented with acute mastoiditis as the first presentation.

Imaging plays a major role in diagnosing and delineating the extent of the disease and planning for the surgery.^5^ Cholesteatoma in congenital atresia is often overlooked on clinical suspicion only thus imaging plays a vital role in the diagnosis.^6^ In the present case also the plan of mastoid exploration and canaloplasty was made prior to surgery after assessing the extent of the disease in the scan. Diffusion-weighted magnetic resonance and imaging (MRI) has greater accuracy in detecting cholesteatoma.^7^

Transmastoid approach was used to remove the cholesteatoma of the temporal bone. Caughey, et al.^8^ have reported a case of canal atresia with congenital cholesteatoma medial to the atretic plate, with intact ossicular chain. They had removed the malleus and incusfor complete removal of cholesteatoma and hearing reconstruction was done using partial ossicular replacement prosthesis. However, our case showed no malleus-incus complex most likely eroded by cholesteatoma and thus temporalis fascia was placed on the head of the stapes for hearing reconstruction.

Similar to our study, Mazita, et al.^2^ reported that in patients with canal atresia associated with cholesteatoma, the hearing outcome is of secondary importance. The primary aim of these patients is to complete cholesteatoma removal, in order to prevent recurrence and complications. Close follow- up is needed in our patient mainly to ensure the canal patency.

Congenital cholesteatoma in a case of aural atresia needs a high degree of suspicion if the patient has facial paresis and progressive hearing loss. Preoperative imaging of the temporal bone aids a lot in assessing the extent of the disease and planning the surgery accordingly. Careful surgical planning is very important to avoid injury to any adjacent tissues and structures. Patients need close and frequent follow up especially if canaloplasty has been done.
